# Management of Concomitant Intrusion and Complicated Crown-Root Fracture Injury of Maxillary Central Incisors in a Child

**DOI:** 10.1155/2023/8750942

**Published:** 2023-11-30

**Authors:** Tariq Abu Haimed, Samah S. Abdeltawab, Rayyan A. Kayal, Mona H. Almotairi, Khalid H. Zawawi

**Affiliations:** ^1^Department of Restorative Dentistry, Faculty of Dentistry, King Abdulaziz University, Jeddah, Saudi Arabia; ^2^Department of Endodontics, Faculty of Dentistry, King Abdulaziz University, Jeddah, Saudi Arabia; ^3^Department of Periodontics, Faculty of Dentistry, King Abdulaziz University, Jeddah, Saudi Arabia; ^4^Department of Orthodontics, Faculty of Dentistry, King Abdulaziz University, Jeddah, Saudi Arabia

## Abstract

Dental intrusions are a severe type of injury because they impact the neurovascular supply of the tooth as well as the supporting tissues which predispose the tooth to necrosis and root resorption. Management of these injuries requires repositioning of the teeth under close monitoring to avoid complications. The management becomes more comprehensive when an intrusion is combined with other injuries, such as a crown-root fracture. This case report represents a 4-year follow-up of a child who suffered from a concomitant injury of intrusion and complicated crown-root fracture to the maxillary immature permanent central incisors. The management involved a multidisciplinary approach including endodontics, pedodontics, orthodontics, periodontics, and prosthodontics. Given the guidelines of dental trauma and the circumstances of the case, the fractured teeth were root canal treated, filled with a bioceramic plug and gutta-percha, and then restored with posts/cores and temporary crowns. The intrusion was managed initially by passive eruption followed by an active orthodontic eruption, after which the teeth were restored with permanent ceramic crowns. Throughout the course of treatment, the teeth showed no complications of root resorption or ankylosis, although one tooth developed a periapical infection which was managed by apical surgery. At the 4-year follow-up, the teeth revealed healthy periodontium and good esthetics.

## 1. Introduction

Dental traumatic injuries (DTIs) are common in children and young adults, accounting for 5% of all body injuries [1, 2]. Boys between the ages of 6 and 12 years are more susceptible to injuries resulting from accidents due to various kinds of physical activity, with more than 90% of the traumas affecting the maxillary incisors [2, 3]. Thus, dealing with DTIs in children is challenging considering the patient's age and the esthetic and psychological impact of the trauma.

Dental traumatic injuries can be classified into teeth and supporting structure injuries [1]. Among the least common types of DTIs are intrusive luxation and complicated crown-root fractures. The incidence of intrusion is less than 2%, while crown-root fractures are estimated at 0.6-5% of all DTIs [2–4]. Cases with intrusive injuries concomitant with complicated crown-root fracture injuries are rarely seen. About 4.7% of intrusion traumas are associated with complicated crown-root fractures [3].

Intrusion is defined as the apical displacement of the tooth into the alveolar bone. The injury impacts all tooth housing tissues including the gingiva, periodontal ligaments (PDL), cementum, and bone as well as the neovascular supply. The outcome of intrusion is considered successful if the tooth returns to its original position without loss of pulpal vitality or damage to the periodontium [1]. However, because of the complex nature of the injury, complications are often inevitable and may include pulp necrosis, inflammatory root resorption, replacement root resorption, and bone loss [4–10]. The multitude of complications occurring depends on several preinjury and injury factors. The stage of root development, severity of intrusion, concomitant injuries, and treatment methods were identified as confounding factors [4–7]. Better prognoses are associated with immature permanent teeth compared to mature permanent teeth as well as teeth with mild intrusion (less than 3 mm) compared to severe intrusions (extending more than 7 mm) [4–9, 11].

When studying the relationship between the outcome and treatment methods of intruded immature teeth, the least complications are achieved with passive or spontaneous eruption [6, 8, 9, 11]. Active eruption by orthodontics or surgical repositioning could be implemented if a spontaneous eruption did not occur or the tooth was subjected to severe intrusion [1, 5, 12]. Orthodontic and surgical repositioning have shown controversial outcomes [6, 8, 9].

Complicated crown-root fractures are defined as trauma that involves the enamel, dentin, and cementum with pulpal exposure. In these injuries, the subgingival extension of the fracture creates inaccessible pathways between the oral cavity and the pulp, which may result in pulpal infection [2, 4, 13, 14].

The treatment of complicated crown-root fractures is aimed at sealing the exposed pulp and uncovering the sound margins of the tooth structure to receive a restoration on healthy periodontium. A conservative treatment method may include vital pulp therapy and bonding of the fractured tooth. However, severely damaged teeth with subcrestal fractures present clinical difficulties in sealing the exposed pulp while providing an adequate ferrule width for permanent restorations. Therefore, in these cases, further intervention including crown lengthening and orthodontic or surgical repositioning is required followed by restorative treatment [12, 15–17].

The management and outcome of DTIs rely on the extent of the injury. A concomitant intrusion with crown-root fracture injury is expected to have more complications than each injury alone [4, 13, 18], and the management is aimed at minimizing these complications. There are established guidelines for the management of each type of DTI [12]. However, cases with concomitant injuries are subjected to clinical judgment due to a lack of conclusive evidence. Careful diagnosis and establishing treatment goals are critical before starting while monitoring progress is important to assure a favorable outcome which may necessitate modification of the treatment if complications occur [12].

The aim of this report is to present a case of a 10-year-old child who suffered from a concomitant intrusive and complicated crown-root fracture injury of the maxillary central incisors. A multidisciplinary approach including endodontics, pedodontics, periodontics, orthodontics, and prosthodontics was followed to achieve the best outcome possible.

## 2. Case Report

### 2.1. History

**Figure 1:**
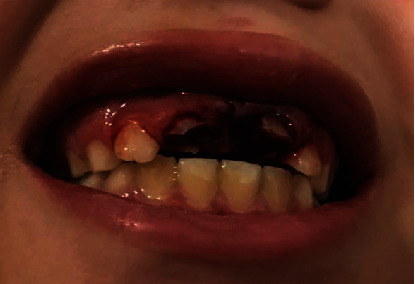
Extraoral picture of the patient at the time of arrival.

A 10-year-old boy presented to the endodontic department at the Faculty of Dentistry, King Abdul Aziz University, roughly 3 hours after sustaining a traumatic injury to his maxillary anterior teeth and upper lip (Figure 1). The trauma occurred after he fell on his face while playing football at school. His medical history was reviewed and found to be insignificant.

### 2.2. Clinical Examination

Upon clinical examination, the patient was noted to have a swollen upper lip and multiple gingival lacerations. Multiple tooth fragments were found to be loosely attached to the gingiva and both maxillary central incisors incurred complicated crown-root fractures during the injury (Figure 1). Intrusion was suspected but could not be confirmed clinically due to the severity of the trauma. The traumatized teeth were tender to percussion and palpation, but no alveolar bone fracture could be detected. The sensibility test revealed vital lateral incisors and primary canines. The lower anterior teeth were unremarkable and showed no injury.

### 2.3. Radiographic Examination

Occlusal radiograph and cone beam computed tomography (CBCT) were taken at the time of the patient arrival [12].

**Figure 2:**
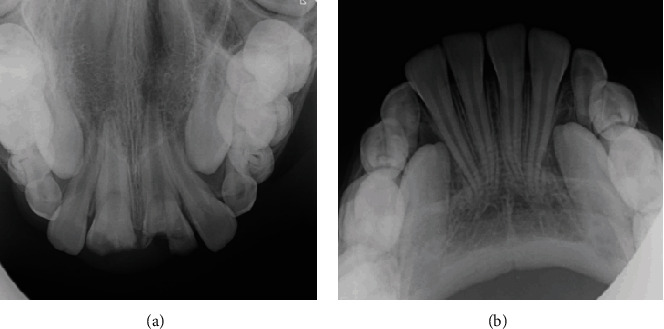
(a) Maxillary occlusal radiograph showing irregular fracture lines of maxillary teeth. (b) Mandibular occlusal radiograph showing normal dentition.

The occlusal radiographs demonstrated irregular fracture lines of teeth #11 and #21 which extended to the pulp. The teeth showed full root length formation with half-open apices [2] (Figure 2).

**Figure 3:**
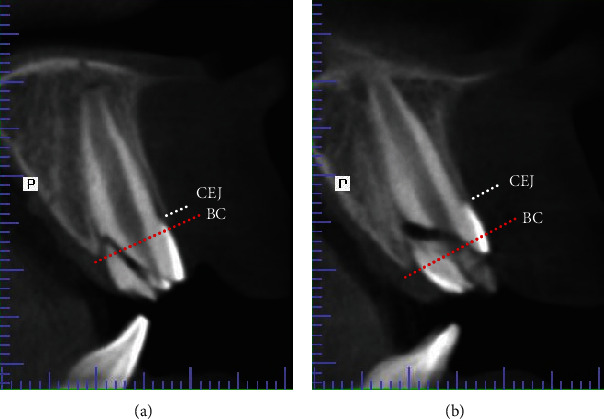
Sagittal CBCT views of (a) #11 and (b) #21 showing multiple fracture lines and loose fragments. From the image scale, the fracture lines extend subcrestally 2 mm in #11 and 5 mm in #21. The dotted lines represent CEJ and bone crest (BC). Therefore, the level of intrusion is approximately 3 mm in #11 and 6 mm in # 21. Root development shows immature apical formation (open apex) in both teeth.

Radiographic interpretation of the CBCT images revealed further details about the extent of the trauma. The sagittal views showed oblique fracture lines extending toward the subcrestal palatal bone approximately 2 mm into tooth #11 and 5 mm into tooth #21 (Figure 3). Additionally, the fracture lines crossed tooth #21 at a steeper angle compared to tooth #11. The severity of intrusion could not be determined from the fractured incisal edge. However, it could be estimated using the cementoenamel junction (CEJ) as a reference point which is placed approximately 1 mm incisal to the bony crest [1]. Adding 1 mm to the current position of CEJ (2 mm #11 and 5 mm #21) indicated an intrusion of approximately 3 mm of #11 (mild intrusion) and 6 mm of #21 (moderate intrusion).

**Figure 4:**
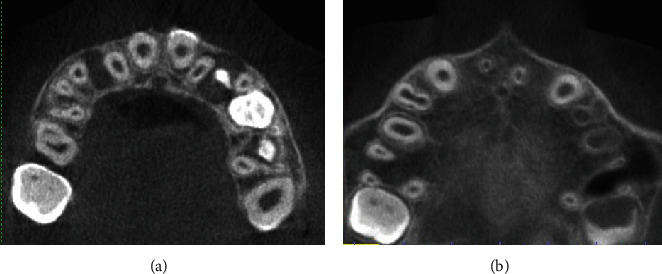
Axial CBCT views showing the position of crowns (a) and roots (b) of #11 in relation to #21 indicating labial intrusion of #21.

The axial views show the labial intrusion of tooth #11 as the position of the crowns and roots of maxillary teeth are compared to each other (Figure 4). However, the buccal and palatal bone plates remained intact.

### 2.4. Diagnosis

Concomitant intrusive luxation with complicated crown-root fracture of maxillary central incisors.

#### 2.4.1. Treatment Procedure

All treatment options were discussed with the patient and his parents, which included either rehabilitation of the traumatized teeth or extraction. The course of treatment was explained, and the risks were outlined. Although saving the teeth would require a lengthy and multidisciplinary approach in addition to the patient's compliance, the decision was made to save the teeth. The patient's parents agreed, and a consent form was signed to initiate the treatment.

**Figure 5:**
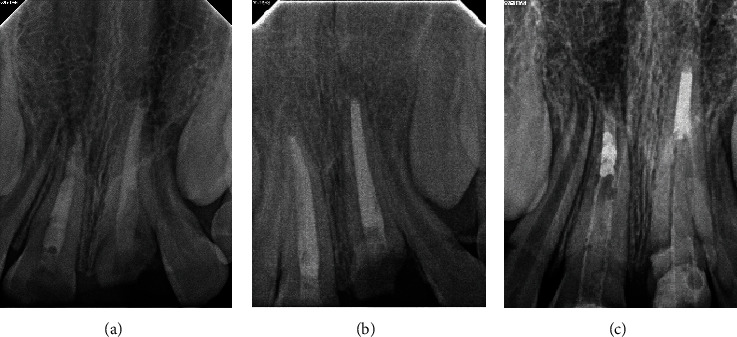
Periapical radiographs of teeth #11 and #21 showing (a) calcium hydroxide paste. (b) Root canal treatment completed. (c) Fiber post and composite core restorations.

Emergency treatment was immediately started to address the wounds and exposed pulp. All loose dental fragments were removed, the gingival lacerations were sutured, and bleeding was controlled. The pulp of the teeth was extirpated, and the canals were irrigated with a chlorhexidine solution followed by an intracanal nonsetting calcium hydroxide paste (Apexit, Ivocalrvivadent, Liechtenstein, Germany) (Figure 5(a)). The working length was taken using an electronic apex locator (Root ZX, Morita, Kyoto, Japan). The teeth were then temporized with glass ionomer restorations (Fuji IX; GC Corporation, Tokyo, Japan). At the end of the emergency visit, a chlorhexidine mouthwash and a regimen of antibiotics (penicillin 500 mg 3 times/day for one week) were prescribed, and oral hygiene instructions were given.

Following the guidelines of intrusive injury, the plan was to allow spontaneous eruption of both teeth under close monitoring followed by orthodontic active eruption of tooth #21. During the second visit (two weeks later), the gingival inflammation subsided, and the root canal treatment was completed. Apical plugs were created using bioceramic putty (EndoSequence, Brasselers, USA), and the canals were filled with thermoplasticized gutta-percha followed by sealing the access with glass ionomer restoration (Figure 5(b)). In the subsequent visit, the teeth were restored with fiber posts and composite cores (Figure 5(c)) to prevent gingival overgrowth and restore the esthetic appearance of the child while waiting for spontaneous eruption.

**Figure 6:**
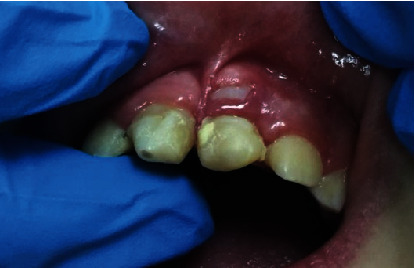
Clinical picture showing eruption of #11 and labial eruption of tooth #21 through the gingiva.

In the following visit (4 weeks), tooth #11 had shown obvious signs of reeruption with 1-2 mm of sound tooth structure can be seen coronal to the gingival margin (Figure 6). However, the labial surface of tooth #21 was noted to be exposed through the labial gingiva. This may be attributed to the labial eruption of tooth #21 coinciding with the direction of the intrusion axis. Additionally, the post/core was slightly mobile.

Therefore, the treatment plan was modified to immediately start orthodontics to adjust the alignment and actively pull the tooth simultaneously.

**Figure 7:**
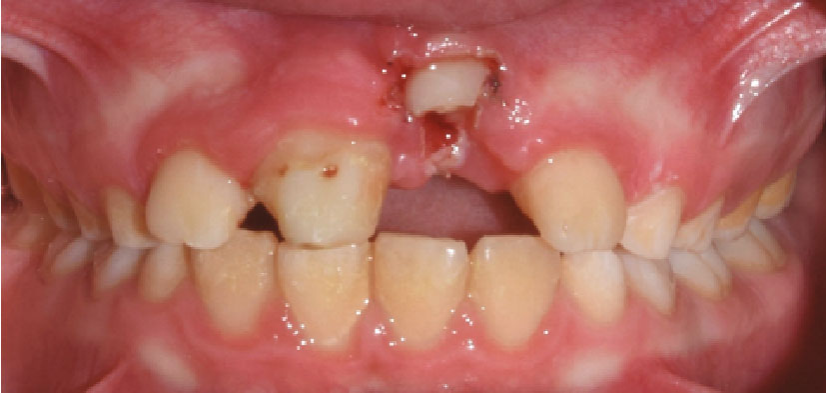
Clinical picture showing tooth #21 after gingivectomy.

The mobile post/core was removed, the canal was filled with gutta-percha, and the tooth was restored with composite extending 4 mm into the canal. A gingivectomy of the labial tissue was then performed with electrosurgery (PerFect TCS, Coltene, Cuyahoga Falls, OH, USA) to expose the sound tooth structure (Figure 7).

**Figure 8:**
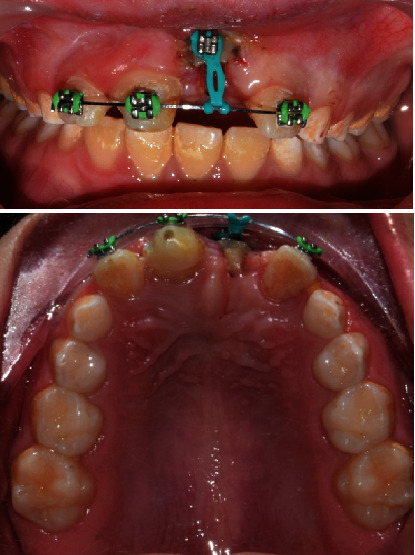
Frontal and occlusal clinical pictures of the teeth after bonding the orthodontic appliance.

The patient was then referred to the orthodontic department for active extrusion. Brackets sized 0.022 slot (OC orthodontics, McMinnville, OR, USA) were passively bonded on the permanent maxillary incisors, and a stainless steel wire size 0.019 × 0.025 was inserted (Figure 8).

**Figure 9:**
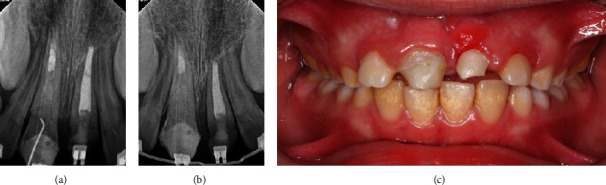
Periapical radiographs showing (a) midway through active orthodontic eruption and (b) after 2-month stabilization before removing the brackets. Tooth #21 shows good crown-to-root ratio after full extrusion. (c) Clinical picture showing the position of teeth after removal of the brackets.

Active extrusion was performed utilizing an elastic chain which exerted approximately 30 g of pulling force. Reactivation by replacing the elastic chain was carried out every 14 days until the desired extrusion was achieved. The total time for extrusion was two months, at which point 1-2 mm of supragingival sound tooth structure was gained (Figure 9). Following extrusion, a stabilization period of 12 weeks was performed to allow bone remodeling.

Examination at this point revealed that tooth #11 showed that both maxillary central incisors had extruded enough to be able to create a ferrule of 1-2 mm. The patient was then referred to the pedodontics department to complete the restoration of tooth #21.

**Figure 10:**
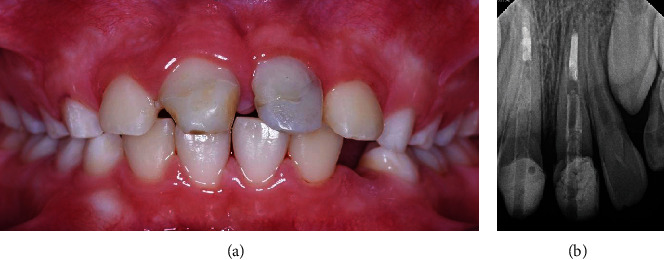
(a) Clinical picture and (b) PA radiograph showing tooth #21 after post and core buildup.

After restoring tooth #21 with a new fiber post and composite core (Figure 10), the patient was scheduled to have the teeth restored with temporary crowns on the next visit. However, the patient failed to return to the clinic for his next visit citing that he was traveling with his family.

**Figure 11:**
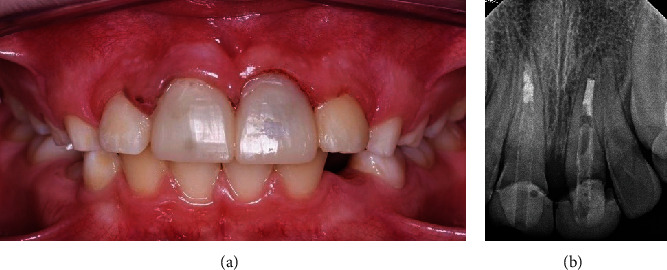
(a) Clinical picture and (b) PA radiograph showing teeth #11 and #21 after temporary crown cementation.

A few months later, the patient returned to the clinic free of any signs or symptoms. The teeth had normal mobility, normal percussion, and normal pocket depths. Given the positive findings, the patient was scheduled to complete his treatment. Teeth #11 and #21 were restored with temporary crowns (Protemp™ Plus, 3M, US), and both the patient and his parents were satisfied (Figure 11).

**Figure 12:**
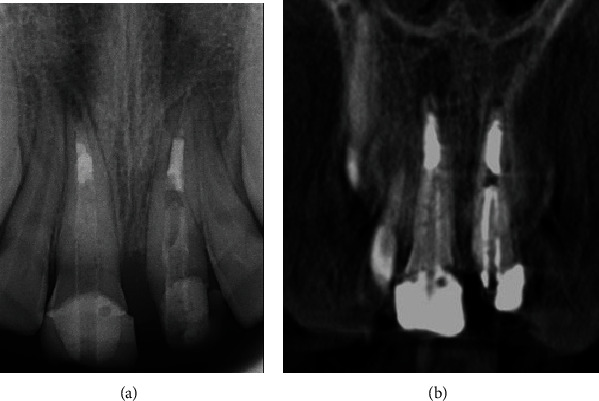
(a) Periapical and (b) CBCT radiographs showing periapical lesion related to tooth #11 and apical closure of tooth #21.

The patient was given an appointment 2 weeks from the temporary placement for the completion of his final ceramic crowns. Unfortunately, this is the time that the country went into lockdown for COVID. The school closed, and all clinics were canceled. This made any follow-up impossible, and we were only able to see the patient approximately one year later, which was 30 months after starting the treatment. At this visit, a complete clinical and radiographic examination was performed and revealed that both central incisors were asymptomatic, there was no pain on percussion or palpation, and the teeth had normal pocket depths. The temporary crown for tooth #21 was missing. Periapical radiographs revealed a periapical radiolucency related to tooth #11 and revealed apical closure of tooth #21. A limited field CBCT confirmed the findings of a periapical lesion about 6 × 3 mm in size related to tooth #11 (Figure 12).

**Figure 13:**
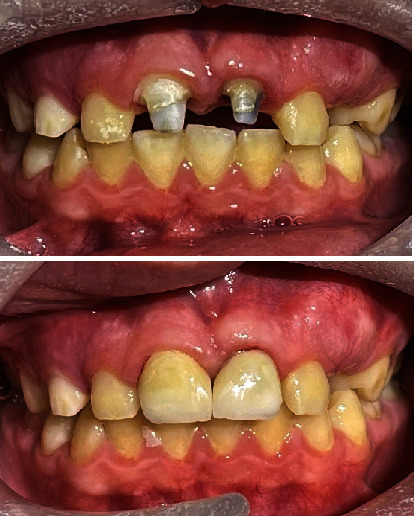
Clinical pictures showing before and after cementation of final ceramic crowns.

To address the periapical pathology, the treatment options of apical surgery or extraction were discussed. The patient and his parents opted to save the tooth but wanted to delay the procedure until the end of the school year. The final restorative treatment was completed and included ceramics crowns (IPS e.max Press, Ivocalrvivadent, Liechtenstein, Germany) in shade A3 which were cemented with resin cement (Multilink Automix, Ivocalrvivadent, Liechtenstein, Germany) (Figure 13).

**Figure 14:**
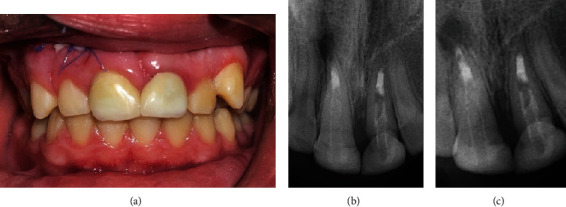
One-week postsurgery follow-up. (a) Clinical image showing healing of the surgery site. Mild gingival inflammation can be seen distal to tooth #21. PA radiographs of (b) before and (c) after surgery.

At the surgical visit, which was just over a year from crown placement, clinical examination showed an adequate thickness of the attached gingiva. A submarginal flap was raised, a minor osteotomy was performed, and the lesion was enucleated. It was noted that the root associated with tooth #11 had an open apex, and the fiber post was extending to the apical third. Root resection was performed followed by retrograde filling with a bioceramic putty (EndoSequence BC Putty, Brasseler, Savannah, GA, USA). Finally, the flap was repositioned and fixed with a resorbable suture. The one-week postsurgery visit showed healing of the surgical site (Figure 14).

It was also noted that there was gingival inflammation around the distal margin of crown #21, which could be attributed to the poor contours of the previously constructed crown (Figure 13).

**Figure 15:**
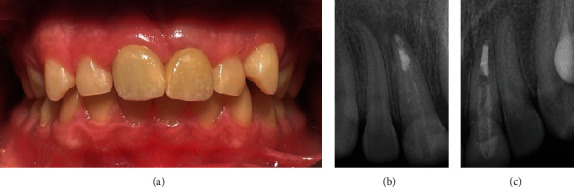
Six-month postsurgery follow-up. (a) Clinical picture showing healthy gingiva and good esthetics. (b, c) PA radiographs showing healing of the surgical site and proper adaptation and proximal contour of the new crowns.

To remedy the tissue health properly, a new set of ceramic crowns was constructed to allow for better hygiene. At six months postsurgery follow-up, progressive healing of the lesion and better gingival health were evident (Figure 15). Although the patient may require orthodontic treatment due to the ectopic eruption of the maxillary left canine, the patient and his parents were satisfied with the entire treatment process. The patient was scheduled for follow-ups every 6 months for the next year and yearly thereafter.

## 3. Discussion

Among DTIs, intrusion and complicated crown-root fracture are the least common and most severe types of luxation and crown fracture dental injuries [2, 4]. A combination of the two injuries represents a real clinical challenge. The literature is very scarce in information regarding the management and outcome of these injuries. Therefore, case reports are a vital source of information.

When facing concomitant injuries, it is essential to recognize the impact of each type of trauma and how the combination of injuries affects the overall outcome. Intrusive injuries directly impact the neurovascular supply of the tooth and surrounding periodontium. In cases of mild to moderate intrusion of teeth that have fully formed roots with open apex, 70%-90% of teeth are likely to develop pulp necrosis [6, 8, 9, 19]. The injured periodontium is susceptible to replacement resorption, while the presence of bacteria intruded from the dental plaque predisposes the roots to inflammatory resorption. The incidence of ankylosis and inflammatory root resorption was estimated to be 20-40% [4, 6, 8–10]. Moreover, marginal bone loss could occur in 0-40% of the cases [8]. An additional injury to intrusion in the form of a crown fracture allows more ingress of bacteria into the root canals, which increases the likelihood of pulpal infection [7, 13, 14, 18, 20]. Wang et al. reported an increase in pulp necrosis from 56% to 76% when intrusion is combined with crown fracture [21]. Hecova et al. showed an increase in pulp necrosis from 3% in teeth which had enamel dentin fractures to 66.6% with concomitant dislocation [4]. Although these studies evaluated crown fractures, similar or worse outcomes may be associated with complicated crown-root fractures. Consequently, the prevalence of infection-related resorption may be increased.

The goal of the management of these injuries is to achieve a favorable healing that includes both the recovery of the pulp and the periodontium under reduced circulation, periodontal damage, loss of tooth structure, and the presence of bacteria [1, 22]. Therefore, clear diagnosis and proper selection of treatment strategy are key components for improving the outcome.

Images such as CBCT are very useful in the diagnosis and selection of treatment of traumatic injuries associated with fractures. Compared to conventional techniques, CBCT images provide a three-dimensional analysis of the injured tooth in relation to adjacent teeth and the surrounding bone structure. They can also help in detecting the presence, extent, and location of crown and root fractures [23]. In the present case, CBCT sagittal images showed fracture lines extending apical to the bone crest about 2 mm in tooth #11 and 5 mm in tooth #21 (Figure 3). The depth of the fractures was a consequence of their pattern and the severity of intrusion. If the teeth were allowed to erupt passively to their original positions, the fracture line of tooth #11 would be about 1 mm supra crestal, while its pattern could be utilized as a finish line for the future prosthetic crown. However, the fracture line of tooth #21 would be located at the bone crest with a relatively horizontal fracture line, thus requiring further intervention to enable the construction of a prosthesis without violating the periodontal health.

After using CBCT imaging to assess the situation, it was possible to come up with treatment options that included immediate active repositioning or spontaneous eruption followed by active repositioning. Due to the nature of the traumatic injury presented, it was opted to allow for initial spontaneous eruption followed by active repositioning of tooth #21 to avoid any additional trauma that would be seen with immediate surgical or orthodontic active repositioning. This suggested strategy would minimize the complications associated with active repositioning and benefit from the fact that spontaneous eruption has the least complications. As spontaneous eruption was occurring, root canal therapy was performed.

Although it is recommended to save the vitality of immature intruded teeth to allow root development, when the risk of developing infection-related resorption outweighs the benefits of preserving the pulp vitality, such as in cases with concomitant injury, immediate commencement of root canal treatment could be performed [6, 13, 19]. Abbott recommended preventive root canal treatment when pulp healing is unlikely to occur to prevent infection-related root resorption resulting from damage to the cementum and PDL [22]. Moreover, restoring the esthetics upon waiting for spontaneous eruption required the root canal space as a source of retention, which served both the temporary as well as final restorations.

Root canal treatment of immature teeth involves managing the apex with apexification or apical plug. Conventionally, apexification is performed by filling and refilling the canals with calcium hydroxide for several months. However, immature teeth with thin dentinal walls are prone to root fracture with long-term calcium hydroxide treatment [24]. With the introduction of bioceramics (calcium silicate cements), it was possible to perform this procedure in one visit and benefit from their superior mechanical and biological properties, including good sealing ability, antibacterial effect, biocompatibility, and the ability to induce hard tissue deposition when in contact with living tissues [25, 26]. Literature shows that bioceramic sealers and mineral trioxide aggregate (MTA) had similar or even better success compared to calcium hydroxide in the formation of apical closure; however, bioceramics do not break down requiring replacement [24, 27, 28]. In the current case, the teeth had full-length roots with thick dentinal walls and half-open apex. An apical plug was the appropriate method of treatment. Therefore, the intracanal calcium hydroxide medication was left for two weeks for disinfection of the root canals followed by a bioceramic plug as a definitive treatment. Pradhan et al. reported that using calcium hydroxide for 2 weeks followed by an MTA plug allowed completion of the treatment in 0.75 months compared to 7 months with calcium hydroxide alone with similar success rates [29]. At 24 months follow-up, tooth #21 showed apical closure while tooth #11 failed due to the development of apical infection.

Spontaneous eruption can be observed within 2-4 weeks and takes up to several months period for completion, which was observed in the present case as both teeth showed eruptive movement after root canal treatment in agreement with previous reports which showed that spontaneous eruption was facilitated after root canal treatment [30, 31]. However, tooth #21 had emerged in a labial direction, primarily due to the path of intrusion (Figure 3). At this stage, active repositioning was initiated to specifically guide the tooth into the desired position.

The second stage of the eruption was performed with active orthodontic extrusion. Besides being a more conservative approach than surgical repositioning, orthodontics was utilized to adjust the alignment of the labially erupting #21. Moreover, it has been reported to provide better esthetics with fewer complications [8, 9, 32]. Typically, active orthodontic eruption is accomplished in 1-3 months at a rate of 1-1.5 mm/week [33]. The higher-than-normal orthodontic forces applied to the teeth are transmitted to the surrounding bone and gingiva at a faster rate than what is required for bone remodeling. Consequently, the teeth are elevated with the gingiva in the form of an increased width of the attached gingiva. Following the orthodontic eruption, a stabilization period of 8-12 weeks is recommended to allow remodeling of the bone and supporting structure and prevent relapse [8]. In the current case, tooth #21 had a good crown-to-root ratio, and therefore, it was possible to orthodontically extrude it about 4-5 mm in addition to that achieved during spontaneous eruption for a total of 6-7 mm. A stabilization period of 3 months was followed to ensure bone remodeling.

One clinical challenge encountered after repositioning is the construction of a restoration that is functionally and aesthetically acceptable. With the progression of the eruptive movement, a smaller diameter of the root is exposed while the mesiodistal width of the original crown space is unchanged. A restoration that is placed on the root must have a relatively large taper to occupy this space. Thus, a restoration with proper contours should be designed to avoid creating areas that are difficult to maintain and ultimately cause gingival inflammation [33]. In the present case, it was noticeable that the distal marginal gingiva of tooth #21 showed inflammation (Figure 14). The patient was informed to pay more attention to this area. Additionally, a new set of crowns with better anatomically contoured margins were delivered, which allowed for better gingival health and esthetics (Figure 15).

An important requirement of a multidisciplinary treatment approach is patient compliance to assess treatment progress and the development of complications. For intruded immature teeth, most pulp necrosis and infection-related root resorption occur within a few months up to 1 year while replacement resorption (ankylosis) may be detected within 1-3 years [4, 5, 7, 9, 19]. In the present case, up to 48-month follow-up, the teeth had shown no signs of resorption, which may be attributed to the early pulp extirpation which in turn prevented infection-related resorption [22]. Additionally, the combined eruption strategy with spontaneous eruption followed by early orthodontic active eruption may have minimized the trauma on the teeth and therefore prevented ankylosis and bone loss following the theory that spontaneous eruption allowed initial healing of the traumatized tissues while early active orthodontic repositioning allowed separation of the tooth from the bone to facilitate cemental healing [7, 8]. Studies by Al-Badri et al. and Tsilingaridis et al. [5, 9] showed that only apical development and degree of intrusion significantly affected root resorption regardless of the treatment method. Therefore, the successful outcome in the present case may be explained by the simple fact that the teeth were immature and suffered mild to moderate intrusion.

There are complications and unforeseen problems that may need to be addressed along the way. In the present case, at the 3-year follow-up visit, periapical radiographs showed an apical lesion related to tooth #11 before the final crowns were cemented. This particular posttreatment disease is related to endodontics rather than directly to the trauma. Failed endodontic treatment may appear years after root canal treatment due to failure to clean an infected canal or because of reinfection [34]. The fiber post of tooth #11 was seen extending unnecessarily to the apical third of the canal which may have contributed to the reinfection during placement [35].

The apical pathology was addressed with surgical intervention. Due to the esthetic positioning of the tooth and the presence of thick attached gingiva, a submarginal flap design was chosen to avoid gingival recession. Postoperative evaluation showed healing of the gingiva and maintenance of the gingival form.

Lastly, the restorative aspect of the case is completed to ensure the healing and management of the case. Since the initial crowns were overcontoured, causing gingival inflammation distal to tooth #21, a new final set was completed to alleviate any chronic gingivitis. At six months postsurgery, follow-up showed better gingival health and healing of the periapical lesion. However, the patient may still require future orthodontic treatment due to the ectopic eruption of tooth #23. At this stage, the patient and the parents were satisfied with the outcome. The patient was scheduled for biannual follow-up for one year followed by annual follow-ups.

## 4. Conclusion

This case represents the management of a concomitant intrusion and complicated crown-root fracture injury. The report showed the progress of treatment up to 4 years follow-up. It may be concluded that concomitant intrusion and crown-root fractures could be managed successfully by spontaneous eruption and orthodontic active eruption. Early root canal treatment may have a positive impact on the outcome by reducing infection-related root resorption. However, it should be mentioned that one of the major limitations in the treatment of the current cases is patient compliance, which was a factor in delaying the treatment on multiple occasions.

## Data Availability

The data will be available on request.
